# Peyronie’s Disease Presenting as Curvature of the Penis: A Case Report

**DOI:** 10.7759/cureus.32055

**Published:** 2022-11-30

**Authors:** Suruchi Dhawan, Avinash Dhok, Suresh Phatak, Kajal Mitra, Ameen Ansari

**Affiliations:** 1 Radiodiagnosis, NKP Salve Institute of Medical Sciences (IMSRC) and Lata Mangeshkar Hospital, Nagpur, IND

**Keywords:** peyronie disease, b-scan ultrasonography, real- time elastography, calcific plaques, erectile dysfunction

## Abstract

Peyronie's disease (PD) usually affects middle-aged men. This condition is characterized by fibrosis and plaque in the tunica albuginea that results in deformity of the penis and makes sexual intercourse difficult. We report a case of a 52-year-old male who presented with complaints of curvature of the erect penis and erectile dysfunction. Based on our imaging findings, the patient was diagnosed with PD. ultrasonography (USG), elastography, and CT findings are described.

## Introduction

Peyronie's disease (PD) is characterized by penile deformity caused by fibrous tissue plaques within the tunica albuginea of the corpora cavernosa of the penis [1]. The condition of erectile dysfunction makes it difficult to engage in sexual activity, and erection is sometimes accompanied by excruciating discomfort [2].

A clearly palpable tunica albuginea plaque that is easily distinguishable from the induration of the cavernosal tissue is seen in the majority of patients with PD. However, imaging is frequently needed to support the clinical diagnosis, assess the severity of the condition, and choose the best course of action for the patient. When evaluating patients with painful penile induration, ultrasonography is frequently the first imaging technique employed in conventional clinical practice [3]. Management of PD ranges from conservative management to surgical treatment [4].

## Case presentation

Patient information:

A 42-year-old male presented to the outpatient department with chief complaints of curvature of the erect penis and erectile dysfunction for one year. There was no history of any associated trauma or urethral instrumentation.

Clinical features

**Figure 1:**
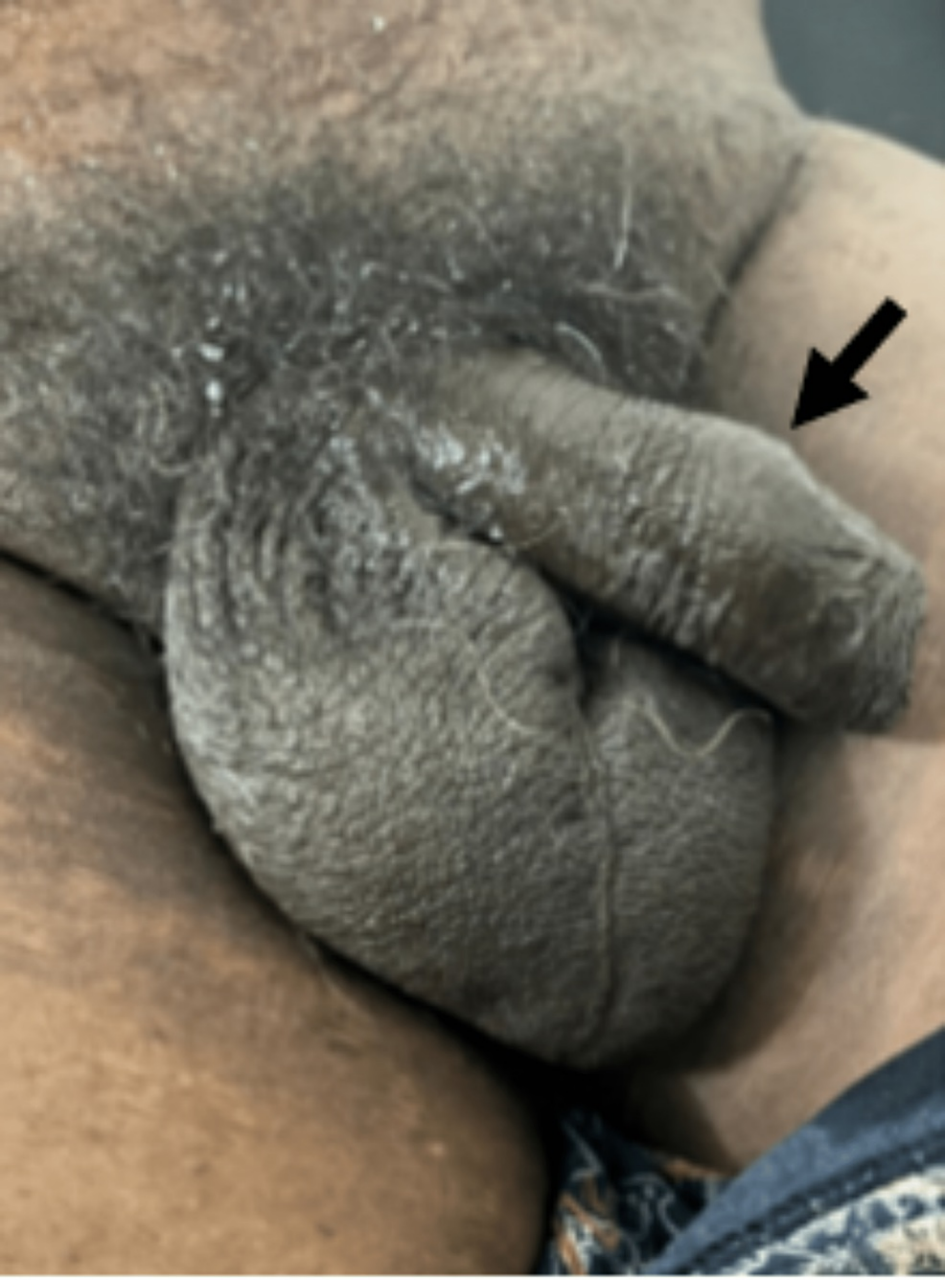
Clinical image depicting curvature of the shaft of the penis

On clinical examination, a curve in the distal end of the penis was noted (Figure 1).

A surgical opinion was obtained and the patient was provisionally diagnosed with PD. The patient was advised penile ultrasound.

Diagnostic assessment

**Figure 2:**
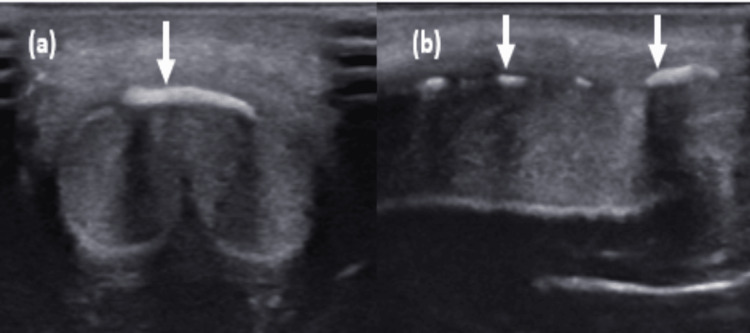
(a) Axial and (b) longitudinal B-mode ultrasound images showing linear calcific plaques along the dorsal aspect of the shaft of the penis

On B-mode ultrasound examination, a few linear calcific plaques having posterior acoustic shadowing were noted along the dorsal aspect of the shaft of the penis and involving the anterior aspect of the corpora cavernosa muscle (Figure 2a and Figure 2b). Along the dorsal side of the penis, fibrotic tissue was observed.

**Figure 3:**
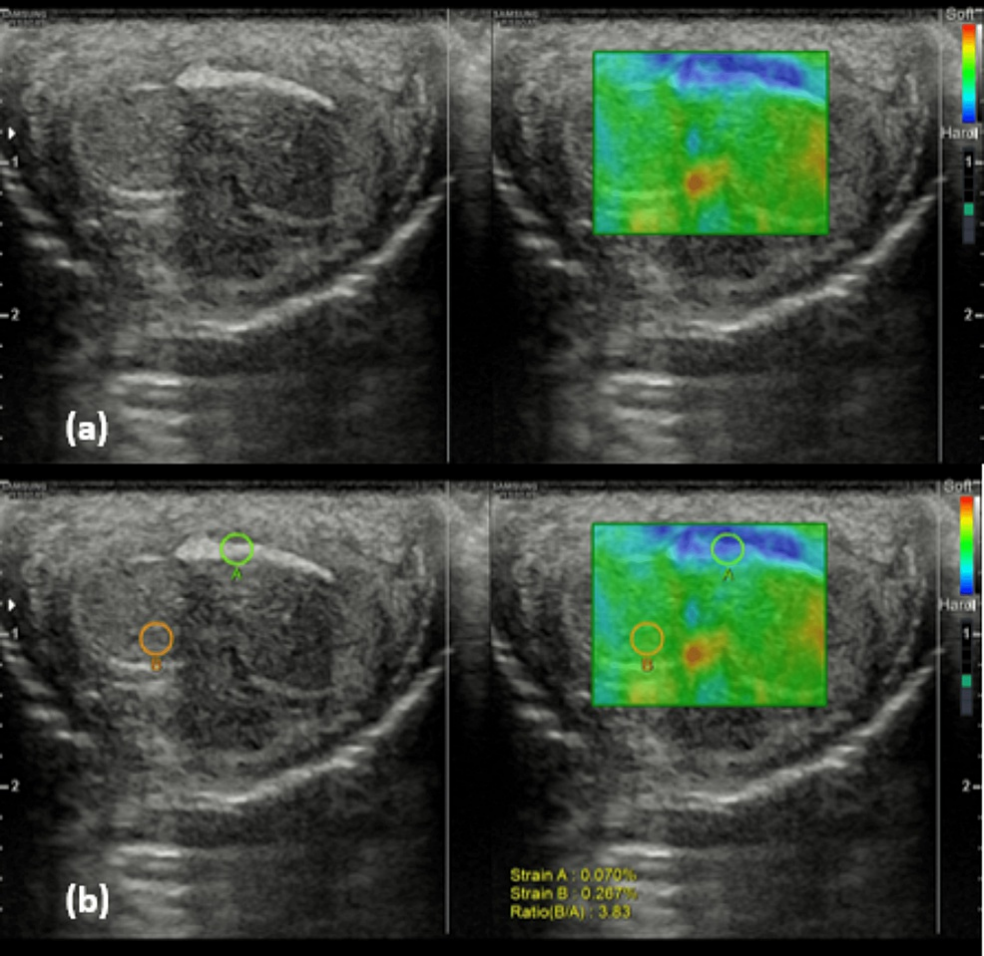
Strain elastography images showing (a) hard plaque depicted by blue color and (b) strain ratio of 3.8 indicating stiffness

Additionally, on ultrasound strain elastography, a strain ratio of 3.8 was noted, which indicated dark blue color suggestive of increased stiffness (Figure 3a and Figure 3b).

**Figure 4:**
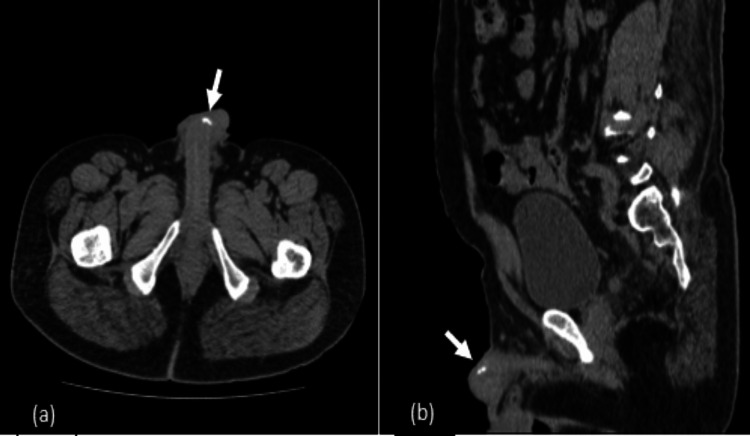
(a) Axial and (b) Sagittal CT images showing calcific plaque along the distal aspect of the shaft of the penis

Further evaluation using CT was done to evaluate the exact location of the plaques and for other deeper plaques that could be missed on ultrasound. CT revealed calcified plaques along the distal end of the shaft of the penis, which further directed us closer to our diagnosis (Figure 4a and Figure 4b).

Based upon the above imaging features, a diagnosis of Peyronie’s disease was made.

Therapeutic interventions

The patient was started on intralesional interferon alfa-2b injection and the patient responded well. Antibiotic coverage (cephalosporine) was given to prevent any secondary infection.

## Discussion

Andreas Vesalius and Gabriele Fallopio, who were famous anatomists initially reported PD, also known as "Induratio penis plastica," but François de la Peyronie clinically classified it as a disorder causing anejaculation in 1743 [1].

Peyronie's disease (PD) leads to the inability to perform penetrative intercourse and related emotional distress [5]. It affects middle-aged men and has a reported age range of 20 to 70 years. PD is primarily due to trauma to the tunica of the penis during erection or semi-erection. This injury triggers an inflammatory response, which causes collagen I and II, fibrin, and calcium to be deposited in the tunica albuginea. Vasogenic erectile failure also occurs due to the plaques obstructing distal flow in the corpora cavernosa. Venous leakages are also a feature of PD. The underlying pathogenesis is thought to be a vasculitis of the sub-tunica connective tissue [6].

USG, MRI, CT scans, radiography, and penile color Doppler and elastography are imaging techniques that aid in the diagnosis and treatment of PD [1].

Due to its accessibility, low risk, and capacity to scan and quantify both the calcified and soft tissue components of PD, US is the preferred imaging technique. If necessary, the vascular state can also be evaluated. Additionally, it identifies tiny lesions that are non-palpable and can also assess the degree of fibrosis [5]. When performing preoperative planning for surgical management, penile Doppler ultrasound, a non-invasive imaging method, is used to assess the shape and vasculature of the penis [7].

Sono-elastography is another modality that helps evaluate non-palpable lesions that cannot be seen on ultrasonography. Real-time strain elastography helps evaluate the elasticity of tissue by applying compression to the tissue and measuring the differences in pre and post-compression vibration. Shear-wave elastography identifies the place of maximal curvature and a region of enhanced tissue stiffness that can be utilized to target intralesional injection treatment [8].

CT is useful, and it is possible to clearly evaluate the calcified plaques; however, CT is not useful in clinical practice and should only be used for research owing to the cost of the examination and the radiation exposure [2].

MRI is useful in determining the location, size, and involvement of other tissues like the corpora and septum. It can also be used to make a diagnosis and choose a course of therapy. The low sensitivity for calcified visualization and the high cost, time, and resource requirements of the MRI are its limitations [2].

Overall disappointing results of medications used in the treatment of Peyronie's disease have been noted. Anti-inflammatory drugs are used in conservative management; they help relieve the pain but do not treat the deformity. Intralesional collagenase injection corrects the deformity, though it is more costly. Significant bending or shortening of the penis with sexual difficulty or partner discomfort are indications for surgical correction of penile curvature [7].

## Conclusions

Numerous imaging modalities are used in evaluating PD but ultrasonography is the modality of choice since it is non-invasive and determines the severity of erectile dysfunction. Additionally, it identifies the location, thickness, and size of the plaque and can also detect calcification within the plaque. Ultrasonography along with clinical history and examination in the form of penis palpation directs toward the diagnosis of PD. This information is sufficient to guide the surgeon in the management of the disease.
